# Interscan reproducibility of cardiovascular magnetic resonance myocardial perfusion reserve index in women with suspected coronary microvascular dysfunction and no obstructive coronary artery disease

**DOI:** 10.1186/1532-429X-18-S1-P82

**Published:** 2016-01-27

**Authors:** Ahmed AlBadri, Janet Wei, Manish Motwani, Sofy Landes, Galen Cook-Wiens, Michael D Nelson, Puja K Mehta, Behzad Sharif, Debiao Li, Daniel S Berman, Louise E Thomson, C Noel Bairey Merz

**Affiliations:** grid.50956.3f0000000121529905Cedars-Sinai Medical center, Los Angeles, CA USA

## Background

Cardiovascular magnetic resonance (CMR) myocardial perfusion reserve index (MPRI) has recently shown promise for detecting coronary microvascular dysfunction (CMD) in women with signs and symptoms of ischemia and no obstructive coronary artery disease (CAD). Prior CMR studies in CAD populations and in healthy volunteers have shown good intra and interobserver reproducibility for MPRI. However, interscan reproducibility is more variable. If MPRI is to be considered useful for the detection of CMD in women, the interscan reproducibility in this population must also be understood, such that proposed MPRI cut-off thresholds can be appropriately adjusted. Therefore, the aim of this study was to determine the interscan reproducibility of MPRI in women with suspected CMD.

## Methods

Rest/stress perfusion CMR was performed at 1.5T using a standardized protocol in 27 women with signs and symptoms of ischemia and no obstructive CAD on two separate days. Cardiovascular medications were held for 24-48 hours before each scan. The same pharmacological stress agent (adenosine/regadenoson) was used for both scans. MPRI was calculated from time-intensity curves of the whole myocardium and blood pool at stress and rest (CAAS MRV 3.3, Pie Medical Imaging B.V., Netherlands). One experienced observer, blinded to clinical data, performed all measurements. Intra-class correlation coefficients (ICC), coefficient of variation (CoV), and Bland-Altman plots were determined.

## Results

**Figure 1:**
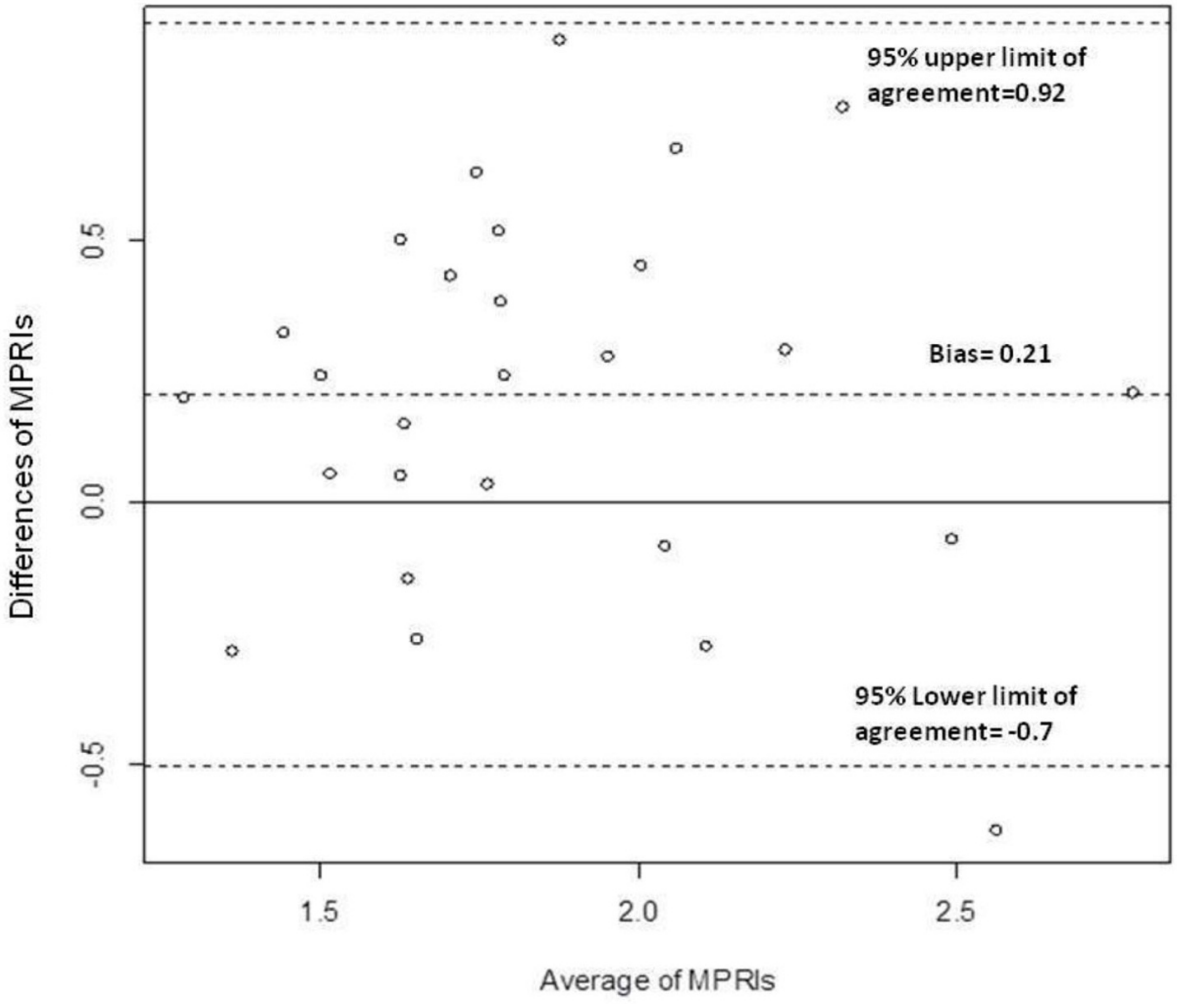
**Bland-Altman plot showing the agreement of whole MPRI between scan 1 and scan 2**. A range of agreement was defined as mean bias ± 2 SD of the mean bias.

Mean age was 51 ± 20 years old and BMI 28 ± 8 kg/m^2^; 55% had hypertension, 7% diabetes, 63% hyperlipidemia and 41% family history of CAD. The time difference between scans was 107 ± 60 days. There was no significant difference between rest or stress hemodynamic variables (summarized as rate pressure product [RPP]) between scan 1 and 2 (rest RPP difference: 82.4 ± 1957.7; stress RPP difference: -1333.8 ± 2812; all p values >0.05). Mean MPRI for the 27 women was higher for scan 2 compared to scan 1 (1.96 ± 0.40 vs. 1.76 ± 0.42); and this relationship persisted even when corrected for resting RPP (1.74 ± 0.5 vs. 1.52 ± 0.35). On Bland-Altman analysis there was a small significant bias for MPRI between scans (bias=0.21 [95% CI: 0.07 to 0.35]) and the 95% limits of agreement were relatively wide (-0.7 to 0.92) (Figure 1). ICC and CoV also indicated only modest interscan reproducibility (ICC 0.541; CoV 23.9%). However, both measures were comparable to values seen in prior studies in CAD populations and healthy volunteers.

## Conclusions

Interscan reproducibility of CMR-derived MPRI in women with suspected CMD was only modest, with relatively wide limits of agreement. This variability is very similar to that seen in other populations and means that some caution must be exercised when using absolute MPRI cut-offs in isolation for the diagnosis of CMD, or repeated measures of MPRI to track response to therapy. The combination of clinical factors and symptom review with MPRI is therefore likely to be the optimal strategy, rather than its use in isolation.

